# Oral care quality improvement intervention results in decreased ventilator associated pneumonia ratio and increased productivity

**DOI:** 10.1186/2047-2994-4-S1-P243

**Published:** 2015-06-16

**Authors:** Y Kenmotsu

**Affiliations:** 1 ICU, Tokai university Hachioji Hospital, Tokyo, Japan

## Introduction

Ventilator associated pneumonia (VAP) is associated with increased morbidity and mortality, increased length of stay, and excess costs. Effective VAP prevention requires multiple interventions, including compliance with an oral care regimen.

## Objectives

A quality improvement (QI) initiative was implemented at a hospital in Japan to assess the effectiveness of a modified oral care regimen on the VAP ratio and caregiver productivity.

## Methods

The QI initiative modified the standard of care for oral care provided to ventilated patients. During the “before” period, standard of care was every 8 hour oral care with toothpaste, toothbrush, and fresh water. During the “after” period, the QI intervention was oral care provided every 4 hours with Q-Care^®^ (Sage Products LLC), consisting of a kit designed for cleaning, debriding, suctioning and moisturizing. Metrics compared included the VAP ratio and time consumption before and after the QI intervention.

## Results

The QI intervention resulted in a 59% reduction in VAP ratio from 2009 through 2011. The standard of care time consumption was 9.8 minutes per oral cleansing, compared with 5.7 minutes per oral cleansing with Q-Care^®^.

## Conclusion

The QI initiative resulted in a decreased VAP ratio and increased productivity.

## Disclosure of interest

None declared.

